# Ising’s Roots and the Transfer-Matrix Eigenvalues

**DOI:** 10.3390/e26060459

**Published:** 2024-05-28

**Authors:** Reinhard Folk, Yurij Holovatch

**Affiliations:** 1 Institute for Theoretical Physics, Johannes Kepler University Linz, 4040 Linz, Austria; r.folk@liwest.at; 2 Institute for Condensed Matter Physics, National Academy of Sciences of Ukraine, 79011 Lviv, Ukraine; 3 𝕃^4^ Collaboration and Doctoral College for the Statistical Physics of Complex Systems, Lviv-Leipzig-Lorraine-Coventry, 79011 Lviv, Ukraine; 4 Centre for Fluid and Complex Systems, Coventry University, Coventry CV1 5FB, UK; 5 Complexity Science Hub Vienna, 1080 Vienna, Austria

**Keywords:** Ising model, Ernst Ising, transfer matrix, Potts model

## Abstract

Today, the Ising model is an archetype describing collective ordering processes. As such, it is widely known in physics and far beyond. Less known is the fact that the thesis defended by Ernst Ising 100 years ago (in 1924) contained not only the solution of what we call now the ‘classical 1D Ising model’ but also other problems. Some of these problems, as well as the method of their solution, are the subject of this note. In particular, we discuss the combinatorial method Ernst Ising used to calculate the partition function for a chain of elementary magnets. In the thermodynamic limit, this method leads to the result that the partition function is given by the roots of a certain polynomial. We explicitly show that ‘Ising’s roots’ that arise within the combinatorial treatment are also recovered by the eigenvalues of the transfer matrix, a concept that was introduced much later. Moreover, we discuss the generalization of the two-state model to a three-state one presented in Ising’s thesis, which is not included in his famous paper of 1925 (*E. Ising, Z. Physik 31 (1925) 253*). The latter model can be considered as a forerunner of the now-abundant models with many-component order parameters.

## 1. Introduction

The now-famous Ising model was suggested by Wilhelm Lenz to his student Ernst Ising in 1922. It was solved in 1D and presented in 1924 in Ernst Ising’s doctoral thesis [1], which was followed by a paper in 1925 [2]. The model was considered by Ising without referring to the Hamiltonian. The latter, in the form as we know it today, was written down by Wolfgang Pauli in 1930 [3]. Instead, Ising defined weights of different configurations and used a combinatorial approach to calculate their contributions to the partition function. In order to proceed, he introduced an auxiliary function, leading to a polynomial whose roots allowed for calculating the partition function. In the thermodynamic limit, one only needs to know the largest root to calculate all thermodynamic functions [4].

Nowadays, it is a textbook exercise to calculate the partition function of a chain of two-state elements with the nearest-neighbor interaction, which we now call a 1D Ising model. Usually, it is performed by applying the transfer matrix to calculate the sum of exponential functions with the Ising model Hamiltonian. The transfer-matrix method was introduced in 1941 by Kramers, Wannier, and Montroll [5,6,7]. The eigenvalues of the transfer matrix allow for obtaining the partition function. The difference in the combinatorial (used by Ising) and the transfer-matrix methods to calculate the partition function lies in the treatment of the Boltzmann weights. Ising concentrates on the Boltzmann weight of a configuration of the whole chain, whereas the transfer-matrix method concentrates on the weights of interacting units.

**Figure 1:**
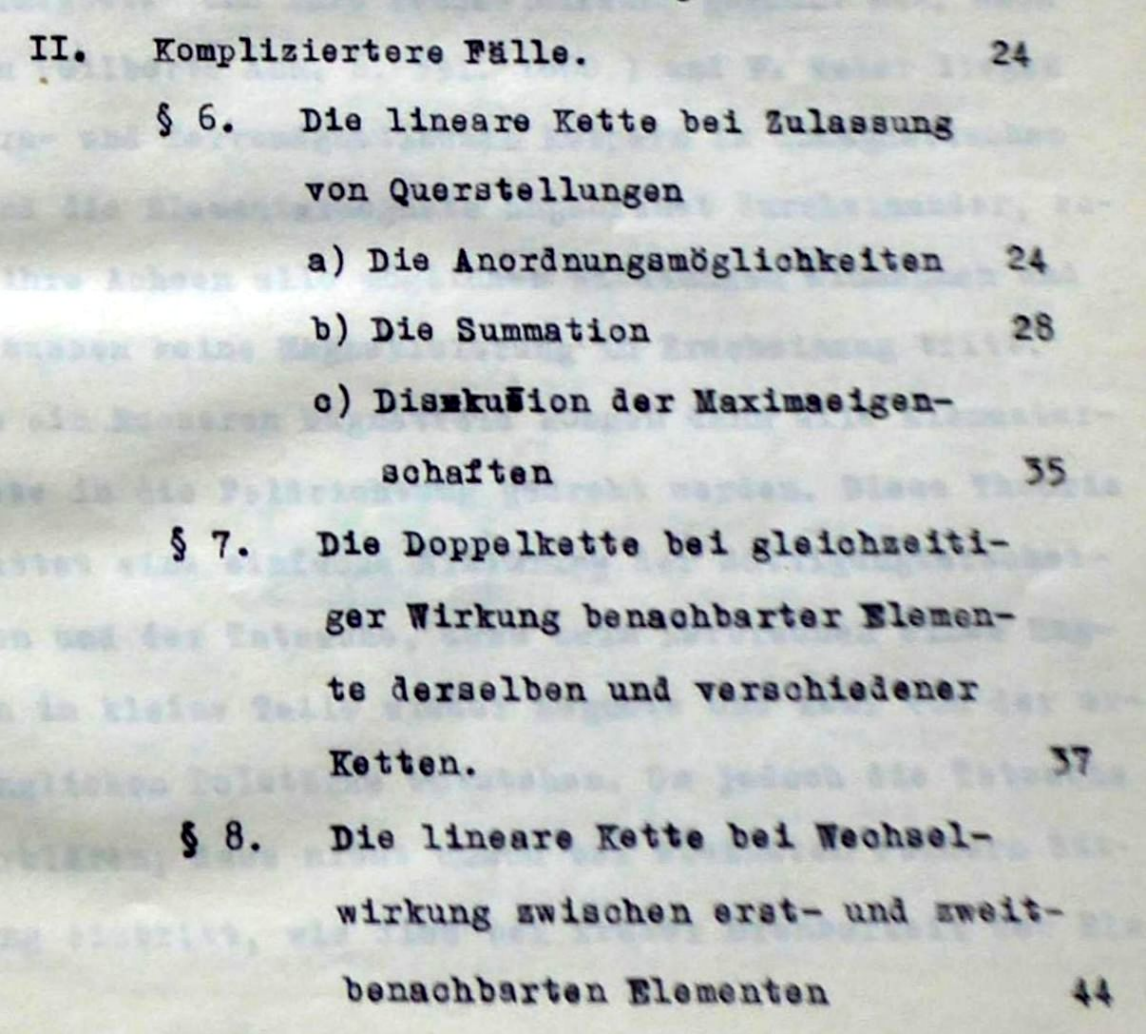
A page from the table of contents of Ernst Ising’s doctoral thesis [1] featuring ‘complicated cases’, Komplizierte Fälle (*Germ.*). One of these cases—The linear chain when transverse positions are permitted (Die lineare Kette bei Zulassung von Querstellungen (*Germ.*))—we discuss in this paper.

The goal of our paper is to attract attention to the important (maybe surprising) point that the polynomial found in the treatment of configurations within the combinatorial approach appears to be the one that follows from the secular equation within the transfer-matrix approach that treats elementary units rather than looking into their configurations. We explicitly show that ‘Ising’s roots’ that arise within the combinatorial treatment are given by the eigenvalues of the transfer matrix. Moreover, we will consider in more detail Ising’s solution for the linear chain with magnetic elements allowing, besides two, parallel and antiparallel, also transverse positions. Although this solution was displayed in Ising’s thesis [1] (see Figure 1), it was not presented in his paper [2]. This is one of the reasons that it is less familiar. In doing so, we will show that Ising’s thesis contained not only the definition of what is called today as the Ising model but also the three-state model, which can be considered as a forerunner to the models with a many-component order parameter, the Potts model being one of them. This extension of the two-state model was solved by Ising, only making assumptions that allowed him to present analytic results. Independent of these approximations, he calculated an exact equation: a polynomial, whose largest root gives the partition function in the thermodynamic limit.

The setup of the rest of the paper is as follows: In Section 2, we will sketch Ising’s solution of the one-dimensional two-state model. Then, in Section 3, we will consider Ising’s solution for the linear chain with magnetic elements allowing also transverse positions. In Section 4, we will show that Ising’s three-state chain (the case when only a single transfer position is allowed) relates to what is currently known as the q=3-state Potts model. The last was introduced much later in 1952 [8], and as far as we know, the fact that its forerunner was solved for d=1 in as early as 1924 has not been acknowledged [9]. We end with conclusions in Section 5.

## 2. Ising’s Method and the Solution for a Two-State Model

As noted in the Introduction, neither the Hamiltonian nor the transfer matrix was used in the original work of Ising; they were not even known(!) (see, e.g., [10,11,12,13] for a more detailed history). In this chapter, we briefly explain the method used in the original publication and show how it relates to the now-standard transfer-matrix technique.

### 2.1. Definition of States, Configurations, and Energy Places

**Figure 2:**
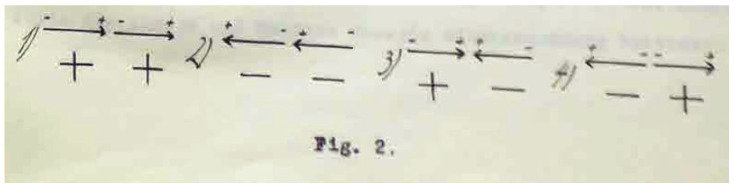
Possible mutual orientations of two neighboring elementary magnets, original figure from Ernst Ising’s thesis [1]. Contributions to energy come from the places where neighboring magnetic moments have opposite orientations, panels 3, 4. Such places are called *energy places—Energiestelle (Germ.)*.

In his thesis, Ernst Ising follows an approach of statistical mechanics, developed at the time by Josiah Willard Gibbs and Ludwig Boltzmann. First, he considers a chain of *N* elementary magnets in an external magnetic field when each of the magnets can be in two states, left/right or plus/minus, as shown in Figure 2. The central quantity of interest that defines thermodynamics of such system in equilibrium is its partition function, defined by the following:

(1) $$Z=\sum_{\left\{\mathrm{configurations}\right\}}e^{-\frac{E}{kT}}$$

where *T* is temperature, and *k* is Boltzmann constant. The sum spans all possible combinations of states of the magnetic elements, i.e., all configurations of the elementary magnets in the chain, and energy E depends on the configuration. It should be noted that energy may have the same value for different configurations.

Ising argues that, for a given configuration of elementary magnets along a chain, contributions to its energy are of different origins: (i) due to the alignment of the magnetic moments along or opposite to the field direction and (ii) due to the mutual orientation of the neighboring elementary magnets. Assuming that the same orientations of neighboring magnetic moments (cf. panels 1, 2 in Figure 2) take minimal energy (chosen to be zero), Ising arrives at the conclusion that only the places where oppositely oriented magnets meet contribute to the energy. He calls such places the *energy places* (Energiestelle) (cf. panels 3, 4 in Figure 2). Consequently, the total energy of a given configuration of *N* elementary magnets is governed by three quantities: number of magnetic moments oriented along (or opposite to) the field, further denoted as $\nu_{1}$ ($\nu_{2}$), and number of energy places σ. The number $N(\nu_{1},\nu_{2},\sigma )$ of different configurations that share the same values of $\nu_{1}$, $\nu_{2}$, and σ defines the degeneracy of a microstate: all such configurations have the same energy. The expression for the partition function (1) of a chain of length *N* readily follows:

(2) $$Z\left(N\right)=\sum_{\nu_{1}+\nu_{2}=N}\sum_{\sigma =0}^{N-1}N\left(\nu_{1},\nu_{2},\sigma \right)e^{-\frac{E_{\alpha }\left(\nu_{1},\nu_{2}\right)+E_{\beta }\left(\sigma \right)}{kT}}\;,$$

with $E_{\alpha }\left(\nu_{1},\nu_{2}\right)/\left(kT\right)=\alpha \left(\nu_{2}-\nu_{1}\right),\;\alpha =\mu h/\left(kT\right)$, $E_{\beta }\left(\sigma \right)/\left(kT\right)=\sigma \beta ,\;\beta =e/\left(kT\right)$; μ and *h* are an elementary magnetic moment and an external magnetic field, and *e* is the energy value of a single energy place. In the notations, Ernst Ising used this in his thesis; this can be rewritten by introducing the Boltzmann weights.

(3) $$A_{1}=e^{\alpha },\;A_{2}=e^{-\alpha },\;B=e^{-\beta }\;.$$

Here, $A_{1}$ and $A_{2}$ are the weights for the states where the elementary magnets are parallel or antiparallel to the external field, and *B* is the weight for the energy place of a neighboring antiparallel elementary magnet. The energy scale was chosen by Ising in such a way that the Boltzmann weight of an energy place for parallel elementary magnets is equal to one. This leads to the following expression for the partition function (2):

(4) $$Z\left(N\right)=\sum_{\nu_{1}+\nu_{2}=N}\sum_{\sigma =0}^{N-1}N\left(\nu_{1},\nu_{2},\sigma \right)A_{1}^{\nu_{1}}A_{2}^{\nu_{2}}B^{\sigma }\;.$$

### 2.2. Ising’s Solution of the Two-State Model: An Auxiliary Function

Ising used methods of combinatorics to explicitly count the number $N(\nu_{1},\nu_{2},\sigma )$, expressing it via the following binomial coefficients (Ising’s method for calculating N was mentioned in Lenz’ review of Ising’s thesis [13] and later cited in 1942 by T. S. Chang, Ph.D., and C. C. Ho, B.Sc., two former students of R. H. Fowler [14]):

(5) N(ν1,ν2,σ=2s+δ)=ν1−1sν2−1s+δ−1+ν2−1sν1−1s+δ−1,

where δ takes on values 0 or 1 depending on the states of the chain boundary elements. Furthermore, in order to proceed, he suggested getting rid of the condition $\nu_{1}+\nu_{2}=N$ in (2) since he was interested in the thermodynamic limit of the partition function. To this end, he introduced the following auxiliary function:

(6) $$F\left(x\right)=\sum_{N=0}^{\infty }Z\left(N\right)x^{N}\;.$$

Given an explicit form for Z(N), Equations (2) and (5), one can perform the summation in (6), arriving at the following:

(7) $$F\left(x\right)=\frac{x[A_{1}+A_{2}-2A_{1}A_{2}\left(1-B\right)x]}{1-\left(A_{1}+A_{2}\right)x+A_{1}A_{2}\left(1-B^{2}\right)x^{2}}\;,$$

simplified as follows:

(8) $$F\left(x\right)=\frac{2x[\cosh\alpha -\left(1-e^{-\beta }\right)x]}{1-2\cosh\alpha \cdot x+\left(1-e^{-2\beta }\right)x^{2}}\;.$$

It is noteworthy that, re-expanding F(x) in terms of *x*, one recovers as expansion coefficients the partition function for finite *N* at free boundary conditions. In the following, the important quantities are the inverse roots $w_{1}$ and $w_{2}$ of the polynomial in *x* in the denominator of Equation (8). Indeed, this function can be put into series with respect to *x* as follows:

(9) $$F\left(x\right)=\sum_{l=0}^{\infty }\left(a_{1}w_{1}^{l}+a_{2}w_{2}^{l}\right)x^{l}\;,$$

with known explicit expressions for $a_{i}$ and $w_{i}$. In particular,

(10) $$w_{1\;,2}=\cosh\alpha \pm \sqrt{\sinh^{2}\alpha +e^{-2\beta }}\;.$$

Observing that $w_{1}>w_{2}$ and comparing Equations (9) and (6), one concludes that the exact result and the leading contribution to the partition function at large *N* are given by the following (the expressions for $a_{1}$ and $a_{2}$ in Equation (11) are given in the thesis and published by Bitter; see Ref. [4], p. 149):

(11) $$Z\left(N\right)=a_{1}w_{1}^{N-1}\left[1+a_{2}/a_{1}{\left(w_{2}/w_{1}\right)}^{N-1}\right]≃a_{1}w_{1}^{N-1}\;.$$

In the thermodynamic limit, this relates the free energy per elementary magnet with $w_{1}$ via the following:

(12) $$F=-kT\lim_{N\to \infty }\ln Z\left(N\right)/N=-kT\ln w_{1}\;.$$

From this, Ising calculated the magnetization of the chain per particle as function of temperature:

(13) $${M=-\partial F/\partial h|}_{T}=\mu \partial \ln w_{1}{/\partial \alpha |}_{T}\;.$$

Substituting (10) into (13), one arrives at the following expression for magnetization that Ising obtained in his thesis:

(14) $$M=\mu \frac{\sinh\alpha }{\sqrt{\sinh^{2}\alpha +e^{-2\beta }}},$$

and that brings about the absence of spontaneous magnetization at any non-zero temperature: M(α=0)=0. A more complete analysis of other thermodynamic quantities using his method can be found in the textbook on ferromagnetism by Francis Bitter [4], who had access also to the thesis.

### 2.3. Reformulation of the Ising Problem with the Hamiltonian and Transfer Matrix for the Two-State Model

For the sake of completeness, let us now briefly summarize the main steps of the transfer-matrix solution of the 1D Ising model [15]. Here, the starting point is the Hamiltonian for the elementary magnets, which, in the meanwhile, have been identified as the following electron spins [3]:

(15) $$H=-J\sum_{j=1}^{N}S_{j}S_{j+1}-h\sum_{j=1}^{N}S_{j},$$

where $S_{j}=\pm 1$ are the spin variables, *h* is an external magnetic field, and *N* is the number of chain sites. This Hamiltonian allows for writing down the energy of a configuration as function of the individual states of the electrons (the former elementary magnets; note that the scale of the magnetic moment has been set to one, μ=1) interacting via exchange interaction [16]. The partition function reads as follows:

(16) $$Z\left(N\right)=\sum_{\left\{\mathrm{states}\right\}}e^{-H/(kT)}=\sum_{\left\{\mathrm{states}\right\}}e^{E_{J}\sum_{j=1}^{N}S_{j}S_{j+1}+\alpha \sum_{j=1}^{N}S_{j}}$$

where $E_{J}=J/\left(kT\right),\;\alpha =h/\left(kT\right)$, and the following sum:

(17) $$\sum_{\left\{\mathrm{states}\right\}}\left(...\right)=\prod_{i=1}^{N}\sum_{S_{i}=\pm 1}\left(...\right)$$

means summation over the spin states on all sites. The expression for the partition function can be written in the form of terms, each depending only on two neighboring spins imposing the following periodic boundary conditions $S_{N+1}=S_{1}$:

(18) $$Z\left(N\right)=\sum_{\left\{\mathrm{states}\right\}}V\left(S_{1},S_{2}\right)V\left(S_{2},S_{3}\right)\cdots V\left(S_{N-1},S_{N}\right)V\left(S_{N},S_{1}\right),$$

with

(19) $$V\left(S,S^{\prime }\right)=e^{\frac{\alpha }{2}S+E_{J}SS^{\prime }+\frac{\alpha }{2}S^{\prime }}.$$

As long as S=±1, $V(S,S^{\prime })$ takes on four values, V(+1,+1), V(+1,−1), V(−1,+1), and V(−1,−1), which can be conveniently represented as the elements of the following so-called transfer matrix:

(20) $$V=\left(\begin{array}{cc} V(+1,+1) & \;V(+1,-1)\\ V(-1,+1) & \;V(-1,-1) \end{array}\right)\;=\;\left(\begin{array}{cc} e^{E_{J}+\alpha } & \;e^{-E_{J}}\\ e^{-E_{J}} & \;e^{E_{J}-\alpha } \end{array}\right).$$

Note that, for the chain with the nearest-neighbor interaction, the dimension of the matrix **V** is defined by the number of states taken by the spin *S*. For the two-state spin variable, the matrix is two by two, and its elements are the Boltzmann weights of all possible configurations of the two neighboring spins. Now, the successive summation over $S_{2},S_{3},\cdots ,S_{N}$ in Equation (18) can be regarded as a successive matrix multiplication. As a result we obtain the following:

(21) $$Z\left(N\right)=\mathrm{Sp}{\left(V\right)}^{N}={\left(\mathrm{Sp}V\right)}^{N}\;.$$

The trace (21) is equal to the sum of matrix eigenvalues, which, for the matrix $V^{N}$, are equal to $\lambda_{1,\;2}^{N}$, with $\lambda_{1,\;2}$ being the eigenvalues of the matrix V (20). The eigenvalues are the solutions of the characteristic (secular) equation of the matrix V.

(22) $$\lambda^{2}-2\lambda e^{E_{J}}\cosh\alpha +2\sinh2E_{J}=0\;.$$

The values readily follow:

(23) $$\lambda_{1,\;2}=e^{E_{J}}\left[\cosh\alpha \pm \sqrt{\sinh^{2}\alpha +e^{-4E_{J}}}\;\right]\;.$$

The transfer matrix’s largest eigenvalue $\lambda_{1}$ defines the thermodynamics of the Ising chain. The corresponding functions are expressed in terms of $\lambda_{1}$ similar to as they were expressed in terms of $w_{1}$ in the former subsection (cf. Equations (11)–(13)). In particular, one obtains the following for the magnetization:

(24) $$M=\mu \partial \ln\lambda_{1}{/\partial \alpha |}_{T}=\mu \frac{\sinh\alpha }{\sqrt{\sinh^{2}\alpha +e^{-4E_{J}}}}.$$

Note the difference in the second terms in the denominators of Equations (14) and (24): $2E_{\beta }$ vs $4E_{J}$. This is due to the fact that the energy gap for the parallel and antiparallel orientations of two neighboring magnetic moments in the original Ising model (Section 2.2) equals *e*, whereas it is equal to 2J for the Hamiltonian (15).

In order to see the agreement with Ising’s result, one has to observe that the corresponding transfer matrix has to be modified as follows:

(25) $$V\;=\;\left(\begin{array}{cc} e^{\alpha } & \;e^{-2E_{J}}\\ e^{-2E_{J}} & \;e^{-\alpha } \end{array}\right)\;=\;\left(\begin{array}{cc} A_{1} & \;B\\ B & \;A_{2} \end{array}\right)$$

since, in Ising’s energy scale, the energy value zero was chosen if the neighboring spins are parallel. The notations of Equation (2) have been used in the second equality to emphasize the appearance of the Boltzmann weights. The characteristic polynomial, therefore, is as follows:

(26) $$\lambda^{2}-2\lambda \cosh\alpha +1-e^{4E_{J}}=0\;$$

or

(27) $$\lambda^{2}-\left(A_{1}+A_{2}\right)\lambda +A_{1}A_{2}\left(1-B^{2}\right)=0\;.$$

These polynomials are to be compared with Equations (8) or (7), which shows that λ can be identified with the inverse roots *w*: $\lambda_{1,\;2}=w_{1,\;2}$.

The exact result for the finite chain reads as follows:

(28) $$Z\left(N\right)=\lambda_{1}^{N}\left(1+{\left(\lambda_{2}/\lambda_{1}\right)}^{N}\right).$$

The difference from Equation (11) is due to the different boundary conditions and diminishes in the thermodynamic limit. It should also be remarked that Ising could not calculate correlation functions since the Hamiltonian and the very nature of interacting elements (spin of the electrons) were found after he finished his thesis (for more on the difference caused by different boundary conditions, see Chapter III in Ref. [17]).

## 3. Ising’s Solution of the Three-State Model

Ising thought that the disappointing result of his search for a ferromagnetic phase in the chain was due to the ‘too great idealization’. However, a calculation of a spatial model within dimension 2 or 3 seemed not to be feasible. Therefore, in the second part of his thesis [1], named ‘Complicated cases’, Komplizierte Fälle (*Germ.*) (see Figure 1), he tried to improve the chain model. In a first step, he enlarged the number of states possible for the elementary magnets by allowing the so-called ‘transverse states’, considering them to be perpendicular to the direction of the up and down states and keeping the nearest-neighbor interaction. Moreover, for symmetry reasons, he allowed *r* different directions of these transverse states (he thought of the sixfold axis of pyrrhotite or the fourfold symmetry axis in magnetite). Since the value of *r* appears in the following calculation only as a trivial parameter, not changing the way of treating the three-state model, from now on, its value is taken as one, r=1.

The way Ising used to calculate the partition function of the three-state model closely follows the steps made for the two-state model. This model now has three energy places describing the energy between neighboring elementary magnets pointing up and down, $e_{12}$; up and transverse, $e_{13}$; and down and transverse, $e_{23}$. Due to the obvious symmetry in interactions, the relation $e_{ij}=e_{ji}$ holds (see also Appendix A). In an external magnetic field *h* corresponding to the up and down direction, the up and down states attain the energy ±μh. In order to prevent the turning of the transverse states in the external field, Ising introduced for them an additional field-independent external energy $e_{e}$.

The partition function is expressed by the number of configurations N and Boltzmann weights analogous to Equations (2) and (3):

(29) $$\begin{array}{ccc} Z\left(N\right)=\sum_{\nu_{1}+\nu_{3}+\nu_{3}=N}\sum_{\sigma_{12}=0}^{N-1}\sum_{\sigma_{13}=0}^{N-1}\sum_{\sigma_{23}=0}^{N-1} & & N(\nu_{1},\nu_{2},\nu_{3},\sigma_{12},\sigma_{13},\sigma_{23})\times \\ & & A_{1}^{\nu_{1}}A_{2}^{\nu_{2}}A_{3}^{\nu_{3}}B_{12}^{\sigma_{12}}B_{13}^{\sigma_{13}}B_{23}^{\sigma_{23}} \end{array}$$

where $\nu_{1}$, $\nu_{2}$, and $\nu_{3}$ are the numbers of up, down, and transverse states corresponding with the obvious condition $\nu_{1}+\nu_{2}+\nu_{3}=N$, and $\sigma_{12}$, $\sigma_{13}$, and $\sigma_{23}$ are the numbers of the respective energy places. The Boltzmann weights read as follows:

(30) $$A_{1}=e^{\alpha },\;A_{2}=e^{-\alpha },\;A_{3}=e^{-e_{e}/\left(kT\right)},\;B_{ij}=e^{-e_{ij}/\left(kT\right)}\;.$$

As before, when the neighbors are in the same states, Ising sets the corresponding energy to zero (the Boltzmann weights are then equal to one).

Following similar steps as described in Section 2 and introducing the auxiliary function to find the partition function, Equation (29), one observes that, again, the denominator of the auxiliary function is a polynomial, now of the third order. The zeros of the polynomial are defined by the following equation:

(31) $$\begin{array}{ccc} & & 1-x[A_{1}+A_{2}+A_{3}]+\\ & & x^{2}\left[A_{1}A_{2}\left(1-B_{12}^{2}\right)+A_{2}A_{3}\left(1-B_{23}^{2}\right)+A_{3}A_{1}\left(1-B_{31}^{2}\right)\right]-\\ & & x^{3}A_{1}A_{2}A_{3}\left[1-\left(B_{12}^{2}+B_{23}^{2}+B_{31}^{2}\right)+2B_{12}B_{23}B_{31}\right]=0\;, \end{array}$$

whereas the maximal solution of this equation defines the free energy in the thermodynamic limit N→∞.

## 4. Transfer-Matrix Formulation for Ising’s Three-State Model

Since the energy of a configuration depends only on the nearest-neighbor states, the transfer matrix can be written down in the same way as before in the two-state model analogous to Equations (20) and (25). Now, instead of a 2×2 matrix, one arrives at a 3×3 matrix with the elements that depend on the nearest-neighbor states:

$$V=\left(\begin{array}{ccc} V(up,\;up) & V(up,\;down) & V(up,\;transverse)\\ V(down,\;up) & V(down,\;down) & V(down,\;transverse)\\ V(transverse,\;up) & V(transverse,\;down) & V(transverse,\;transverse) \end{array}\right)\;.$$

Inserting the corresponding Boltzmann weights, the transfer matrix takes on the following form:

(32) $$V=\left(\begin{array}{ccc} A_{1} & B_{12} & B_{31}\\ B_{12} & A_{2} & B_{23}\\ B_{31} & B_{23} & A_{3} \end{array}\right)\;.$$

The characteristic equation for the eigenvalues of the matrix reads as follows:

(33) $$\begin{array}{ccc} & & \lambda^{3}-\lambda^{2}\left[A_{1}+A_{2}+A_{3}\right]+\\ & & \lambda \left[A_{1}A_{2}\left(1-B_{12}^{2}\right)+A_{2}A_{3}\left(1-B_{23}^{2}\right)+A_{3}A_{1}\left(1-B_{31}^{2}\right)\right]-\\ & & A_{1}A_{2}A_{3}\left[1-\left(B_{12}^{2}+B_{23}^{2}+B_{31}^{2}\right)+2B_{12}B_{23}B_{31}\right]=0\;. \end{array}$$

As in the case of the two-state model, the equation for the transfer-matrix eigenvalues and that for the inverse roots of the three-state model coincide with each other (cf. Equations (33) and (31)). Note that only the energies present in the Boltzmann weights are to be defined to obtain the corresponding eigenvalues for calculating the partition function. In his thesis [2], Ising generalized this method also for the cases of two chains and for a chain with next nearest neighbors (cf. paragraphs 7 and 8 in the thesis table of contents displayed in Figure 1: § 7; the double chain with the simultaneous action of adjacent elements of the same and different chains—Die Doppelkette bei gleichzeitiger Wirkung benachbarter Elemente derselben und verschiedener Ketten (*Germ.*), § 8; and the linear chain at interaction between first and second adjacent elements—Die lineare Kette bei Wechselwirkung zwischen erst- und zweit-benachbarten Elementen (*Germ.*)). The problem for Ising was that, already for the three-state case, the characteristic equation is a polynomial of higher-than-second order, and in order to calculate the partition function in the thermodynamic limit, one has to know its largest solution. In the cases mentioned, this can be performed only by solving the equation numerically.

## 5. Conclusions and Further Developments

In his original approach, Ernst Ising used combinatorial methods to calculate weights of different elementary magnet configurations and their contributions to the partition function. To this end, he used an Ansatz, introducing an auxiliary function, Equation (6), leading to a polynomial whose roots allowed for calculating the system thermodynamics. In particular, the largest root of the polynomial gives an asymptotically exact expression for the partition function. Comparing Ising’s calculation of the partition function with the analysis of the same model by the transfer-matrix technique, a method discovered much later shows that both methods lead to the same characteristic polynomials. In particular, we show in this paper that ‘Ising’s roots’ arising within the combinatorial treatment can be identified as the eigenvalues of the transfer matrix.

In 1974, explaining the beginning of the Potts model, Cyril Domb wrote the following [18]: ‘In 1951 when the present author was at Oxford he pointed out to his research student R B Potts that the transformation discovered by Kramers and Wannier (1941) for the two-dimensional Ising model could be generalized to a planar vector model having three symmetric orientations at angles of 0, 2π/3, 4π/3 with the axis. Hence the Curie temperature could be located for this model. He suggested that it might be possible to extend the result to a planar vector model with *q* symmetric orientations. After a detailed investigation Potts (1952) came to the conclusion that the transformation did not generalize to a planar vector model with *q* orientations, but instead to a *q*-state model in which there are two different energies of interaction which correspond to nearest neighbors being in the same state or different states’.

Perhaps, when it comes to a model with a multi-component discrete order parameter, the Potts model, a brief history of which is sketched above, comes to mind first. However, as we emphasize in this paper, an attempt to increase the order parameter component number was already contained in Ising’s thesis, carried out almost 30 years earlier [1]. These results were not included in his 1925 paper [2] and are therefore less well known. Ernst Ising’s thesis not only analyzes what is called today as the Ising model but also contains the description of the three-state model, which can be considered as a forerunner to the models with a many-component order parameter, the Potts model of 1952 [8] being one of them.

Meanwhile, different variants of *q*-state models have been investigated even for cooperative phenomena outside magnetism (see exercises in Ref. [19], p. 75). For example, the classical spin-1 Ising model is more suitable for describing phase transitions and critical phenomena occurring in physical systems characterized by three states, and such a model was suggested in 1966 by Blume and Capel for magnetic phase transitions [20,21]. Later, in 1971, it was extended by Blume, Emery, and Griffiths and used to describe the phase separation in He3–He4 mixtures [22] (see also [23] for recent discussion). Three-state models are popular in the description of biological, economic, social phenomena. Depending on the phenomenon under consideration, obvious interpretations mean three-state oppositions like buy–sell–hold, susceptible–infected–recovered, and left wing–center–right wing.

At the time of Ising’s thesis, the importance of the spacial dimensionality of a physical system for the existence of a phase transition was not known. A modern understanding of such phenomenon assumes spatial dimensionality, along with symmetry, order parameter component number, and interaction range as key factors determining the class of universality of the system under consideration. Renormalization group theory proved the existence of a lower critical dimension for models within a certain universality class below which no ordered phase is possible at non-zero temperature. However, the transition at T=0 can be regarded as a critical point [24]. Accordingly, for the problem considered by Ising, such a symmetry group is a discrete group $L_{2}$, and the corresponding lower critical dimension is d=1. Therefore, no spontaneous magnetization can be observed for this model at non-zero temperature at d=1—a fact that was confirmed by Ising’s exact solution. An absence of ordering at non-zero temperature for d=1 classical short-range interacting models is attributed to an entropy excess relative to interaction energy. Notoriously, for the d=1 Ising model, the entropy–energy balance can be achieved by considering the so-called invisible states [25] that, under certain conditions [26,27], can lead to entropy decrease and, thus, promote the spontaneous ordering.

This work has been conducted in the frames of a larger project that aims at the bilingual commented publication of Ernst Ising’s doctoral thesis. We deeply acknowledge our long-standing and enjoyable collaboration with Bertrand Berche and Ralph Kenna. We devote this paper to the memory of Ralph Kenna, our dear friend who recently left us, not even reaching his sixtieth birthday.

## Figures and Tables

**Figure A1:**
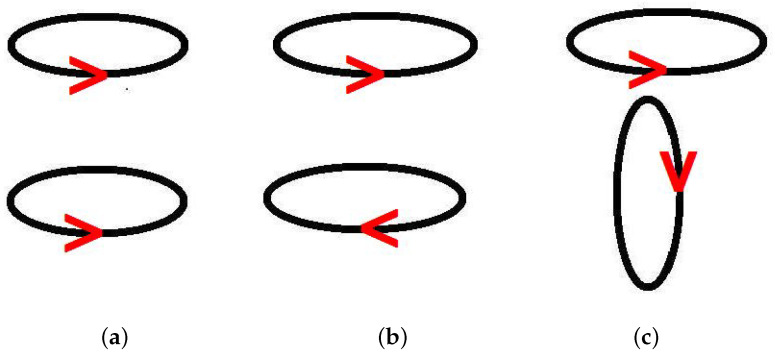
Different configurations of two neighboring rotating electrons (red arrows show direction of rotation). Each of the electrons can be in one of the three states: rotating in a parallel plane clockwise (state ‘+’ or ‘up’), counterclockwise (state ‘-’ or ‘down’), or in a perpendicular plane (state ‘0’ or ‘transverse’). The direction of the chain is downwards and coincides with the direction of the external field if present. Corresponding Boltzmann weights are given in Equation (30). The configuration (**b**) has the weights $B_{12}$ and (**c**) $B_{23}$ (or $B_{13}$, according to Schottky’s calculation).

**Figure A2:**
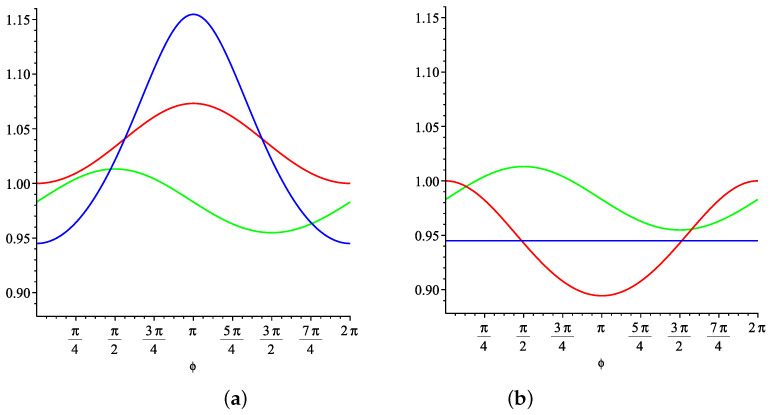
Mean interaction energies of two rotating electrons of different configurations as functions of phases. (**a**) Electrons are placed beside each other; (**b**) electrons are placed above each other. Colors show the states of the electron pairs: both electrons rotate in parallel planes in the same (red) and in opposite (blue) directions; one electron rotates in a parallel plain, and another one in a perpendicular plain (green). Note that, in the latter case, the mean interaction energy does not depend on the rotation direction.

## Data Availability

No new data were created or analyzed in this study. Data sharing is not applicable to this article.

## Appendix A. Schottky’s Idea and Transverse States

At the time when Ising wrote his thesis, not only the model Hamiltonian and the transfer-matrix method were unknown. The very mechanism of interaction leading to the appearance of the low-temperature ferromagnetic phase was also a mystery. The dipole interaction, which was known at that time, is too weak to explain the observed values of the Curie temperature ($T_{c}∼1000$ K for Co, Fe, Ni), whereas the discovery of the exchange interaction, as well as the very concept of spin, was still to come (see Ref. [28] for a more detailed discussion). In the introduction to the thesis, Ising mentions Schottky’s reasoning [29] about a possible physical basis for this interaction. However, a reference to Schottky is absent in Ising’s paper [2] written based on the materials of the thesis. In this appendix, we explain in more detail Schottky’s views as expressed in his paper [29] and show how they may be related to the three-state model considered by Ising.

Schottky formulated his ideas in terms of the old Bohr–Sommerfeld picture of quantum mechanics. He considers circling electrons on neighboring atoms, as shown in Figure A1, panels (a) and (b). Because of the rotation, the electrons produce elementary magnetic moments, pointing along the axis, perpendicular to the rotation plane. Therefore, the direction of the chain of elementary magnets is downwards in Figure A1. When the electrons are circling in the same directions, panel (a), the induced magnetic moments are parallel. The opposite directions, panel (b), corresponds to the antiparallel orientation of the induced magnetic moments. The mean energy of the (electrostatic) interaction *E* depends on the distance *d* between the electrons. The latter, in turn, depends on both the phase ϕ and the direction of rotation. The key concept of Schottky’s theory is a *synchronism* in the motion of the circling electrons: the energy *E* of the two electrons should be as small as possible during the circling around the nucleus. It is the phase between the two circling electrons that is adjusted to minimize the energy of the Coulomb interaction. The preferred, parallel or antiparallel, configuration of magnetic moments is defined by the difference between their electrostatic energies. The difference in the energy due to the magnetic dipole interaction might be neglected, being much smaller than the electrostatic energy difference.

For illustration purposes, as in Ref. [28], let us calculate the distance between two electrons rotating with frequency ω along two circles of radius *r* placed above each other at distance *a* (see Figure A1a). The phase between the two rotations is fixed to ϕ. The coordinates of two electrons read as follows:

(A1)x1=rcosωtx2=rcos(ωt+ϕ)(A2)y1=rsinωty2=rsin(ωt+ϕ)(A3)z1=0z2=a.

Introducing the notation ωt=τ, R=r/a, we obtain the following for the distance if both electrons rotate in the same direction:

(A4) $$d_{1}\left(R,\tau ,\phi \right)=\sqrt{1+4R^{2}\sin^{2}\left(\phi /2\right)}\;.$$

Now changing the direction of the circulation of one electron [τ→−τ ] (cf. Figure A1b), one obtains for the distance the following:

(A5) $$d_{2}\left(R,\tau ,\phi \right)=\sqrt{1+4R^{2}\sin^{2}\left(\tau +\phi /2\right)}\;.$$

Consider now one electron rotating in the xy plane (as in the former case), and the other one rotating in the zy plane, as shown in Figure A1c (the direction of rotation does not matter in this case). The coordinates read as follows:

(A6)x1=rcos(ωt)x2=0(A7)y1=rsin(ωt)y2=rcos(ωt+ϕ)(A8)z1=0z2=rsin(ωt+ϕ)+a.

We obtain for the distance the following:

(A9) $$d_{3}\left(R,\tau ,\phi \right)=\sqrt{2R^{2}+1\pm 2R\sin\left(\tau \right)-2R^{2}\cos\left(\tau \right)\sin\left(\phi \pm \tau \right)}\;.$$

Here, ± signs correspond to different directions of electron rotation. Note, however, that all four combinations of ± signs lead to the same values of the mean energy.

The mean Coulomb energy for different configurations of rotating electrons can be defined as follows:

(A10) $$E_{i}\left(R,\phi \right)=\frac{1}{2\pi }\int_{0}^{2\pi }\frac{d\tau }{d_{i}\left(R,\tau ,\phi \right)}\;.$$

Repeating a similar reasoning, one obtains mean energies for the cases when electrons are circling in the planes beside (and not above) each other. The resulting mean energy plots are shown in Figure A2. As one can see from the plots, the analysis of the three-state model within the framework of Schottky’s synchronism concept leads to a similar conclusion as in the case of the two-state model [28,29]: the existence of configurations characterized by the minimum average interaction energy. In this way, the model still had a weak microscopic (physical?) basis.
